# Suppression of Myeloid Cell Arginase Activity leads to Therapeutic Response in a NSCLC Mouse Model by Activating Anti-Tumor Immunity

**DOI:** 10.1186/s40425-019-0504-5

**Published:** 2019-02-06

**Authors:** Juan J. Miret, Paul Kirschmeier, Shohei Koyama, Mingrui Zhu, Yvonne Y. Li, Yujiro Naito, Min Wu, Venkat S. Malladi, Wei Huang, William Walker, Sangeetha Palakurthi, Glenn Dranoff, Peter S. Hammerman, Chad V. Pecot, Kwok-Kin Wong, Esra A. Akbay

**Affiliations:** 1 Dana Farber Cancer Institute, Belfer Institute of Cancer Science, Boston, MA USA; 2 Department of Respiratory Medicine and Clinical Immunology, Graduate School of medicine, Osaka University, Osaka, Japan; 3 Department of Pathology, University of Texas Southwestern Medical Center, Dallas, TX USA; 4 Simmons Comprehensive Cancer Center, Esra Akbay, PhD, Address: 5323 Harry Hines Blvd, Dallas, TX 75390 USA; 5 Department of Medical Oncology, Dana Farber Cancer Institute, Boston, MA USA; 6 Department of Bioinformatics, University of Texas Southwestern Medical Center, Dallas, TX USA; 7 Bioinformatics Core Facility, University of Texas Southwestern Medical Center, Dallas, TX USA; 8 Elstar Therapeutics, Cambridge, MA USA; 9 Novartis Institutes for Biomedical Research, Cambridge, MA USA; 10 Cancer Program, Broad Institute of Harvard and MIT, Cambridge, MA USA; 11 University of North Carolina Chapel Hill, Lineberger Cancer Center, Chapel Hill, NC USA; 12 Laura and Isaac Perlmutter Cancer Center, New York University Langone Medical Center, New York, NY USA

**Keywords:** Arginase, MDSC, Arginine, Metabolic checkpoint, Aminoacid, Immunocompetent, Autochthonous

## Abstract

**Background:**

Tumor orchestrated metabolic changes in the microenvironment limit generation of anti-tumor immune responses. Availability of arginine, a semi-essential amino acid, is critical for lymphocyte proliferation and function. Levels of arginine are regulated by the enzymes arginase 1,2 and nitric oxide synthase (NOS). However, the role of arginase activity in lung tumor maintenance has not been investigated in clinically relevant orthotopic tumor models.

**Methods:**

RNA sequencing (RNA-seq) of sorted cell populations from mouse lung adenocarcinomas derived from immunocompetent genetically engineered mouse models (GEMM)s was performed. To complement mouse studies, a patient tissue microarray consisting of 150 lung adenocarcinomas, 103 squamous tumors, and 54 matched normal tissue were stained for arginase, CD3, and CD66b by multiplex immunohistochemistry. Efficacy of a novel arginase inhibitor compound 9 in reversing arginase mediated T cell suppression was determined in splenocyte ex vivo assays. Additionally, the anti-tumor activity of this compound was determined in vitro and in an autochthonous immunocompetent Kras^G12D^ GEMM of lung adenocarcinoma model.

**Results:**

Analysis of RNA-seq of sorted myeloid cells suggested that arginase expression is elevated in myeloid cells in the tumor as compared to the normal lung tissue. Accordingly, in the patient samples arginase 1 expression was mainly localized in the granulocytic myeloid cells and significantly elevated in both lung adenocarcinoma and squamous tumors as compared to the controls. Our ex vivo analysis demonstrated that myeloid derived suppressor cell (MDSC)s cause T cell suppression by arginine depletion, and suppression of arginase activity by a novel ARG1/2 inhibitor, compound 9, led to restoration of T cell function by increasing arginine. Treatment of Kras^G12D^ GEMM of lung cancer model with compound 9 led to a significant tumor regression associated with increased T cell numbers and function, while it had no activity across several murine and human non-small cell (NSCLC) lung cancer lines in vitro*.*

**Conclusions:**

We show that arginase expression is elevated in mouse and patient lung tumors. In a KRAS^G12D^ GEMM arginase inhibition diminished growth of established tumors. Our data suggest arginase as an immunomodulatory target that should further be investigated in lung tumors with high arginase activity.

**Electronic supplementary material:**

The online version of this article (10.1186/s40425-019-0504-5) contains supplementary material, which is available to authorized users.

## Background

NSCLC is the leading cause of cancer-related mortality world-wide [1]. Targeted therapies provide initial survival advantage in patients whose tumors harbor targetable genomic alterations, such as *EGFR* mutations and anaplastic lymphoma kinase (*ALK*) fusions [2–4]. However, the majority of lung cancers don’t have a targetable mutation [5]. Evading and escaping the anti-tumor immune responses by immune-suppressive mechanisms is critical for tumor initiation, and growth and is considered to be one of the hallmarks of cancer [6]. These mechanisms include physical and biochemical barriers to T cell infiltration to tumor sites [7, 8], inhibitory cellular interactions [9, 10], and metabolic deregulation [11]. Blockade of the PD-1/PD-L1 inhibitory interaction in particular is less toxic than chemotherapy and has been shown to have clinical efficacy in some lung tumors that cannot be treated with targeted therapies [12, 13]. While there is considerable excitement in implementing immunotherapies, PD-1/PD-L1 blockade is associated with a clinical response rate of only about 20% in unselected NSCLC [14–16]. These findings have supported the premise that evasion of immune destruction contributes to NSCLC pathogenesis and alternative immune evasion mechanisms may operate in PD-1 blockade resistant tumors.

Amino acid metabolism is recognized as a critical immune regulatory control point, which tempers T cell responses through a variety of mechanisms [9, 17]. Tryptophan degradation by IDO1/2 or TDO limits innate and adaptive immune responses by depleting the tumor local microenvironment of trytophan and by promoting the accumulation of kynurenine resulting from catabolism of tryptophan [18]. Genetic ablation of IDO1 has been shown to inhibit the growth of Kras mutant tumors in mouse tumor models associated with an enhanced development of an anticancer immune response [19]. These observations supported the discovery and development of novel IDO1 inhibitors, which reduced tumor growth in preclinical in vivo studies [20]. A phase III trial assessing the efficacy of IDO inhibition in combination with pembrolizumab in melanoma was not successful, highlighting the need to better understand metabolic checkpoint driven immune suppression and the role of other such checkpoints in immunotherapy responsive tumors like lung and melanoma.

Similar to tryptophan metabolism, arginine degradation contributes to T cell suppression [21]. Besides dietary intake, arginine is derived from cellular protein break-down or endogenous de novo arginine production. While most cells are able to synthesize arginine from citrulline through the enzymes arginosuccinase synthase (AS) and arginosuccinase lyase, some cells are not able to produce arginine and are dependent on extracellular arginine making arginine a semi-essential amino acid [22, 23]. In mammalian cells, L-Arg can be catabolized by 4 enzymes:nitric oxide synthase (NOS), arginases I and II, L arginine: glycine amidinotransferase, and arginine decarboxylase. Arginases and NOS are the main enzymes regulating extracellular arginine levels in the tissues. M1 macrophages produce NO via iNOS and M2 macrophages produce ornithine via arginase from arginine [24] . Two mammalian arginase isoforms (ARG1 and ARG2) convert arginine to ornithine and urea; and have distinct tissue, cellular and subcellular distributions [25]. ARG1 is a cytosolic enzyme constitutively expressed in hepatocytes. ARG2 is detected in mitochondria and expressed in kidney, brain, prostate, intestine, and pancreas [24].

To date most of the immunosuppressive functions of arginine metabolism have been associated with elevated ARG1 levels in immunosuppressive myeloid cells but not ARG2 [26]. In mouse, ARG1 level is modulated by several immunosuppressive cytokines (IL-4, IL-10, and IL-13) and prostaglandins (PG2) [27]. In addition, the ARG1 levels can be affected by tumor metabolism; lactic acid produced by tumor cells, a by-product of aerobic or anaerobic glycolysis, up-regulates the levels of ARG1 in tumor associated macrophages by stabilizing hypoxia-inducible factor 1a (HIF1a) [28]. Amino acid sensor, general control nonderepressible 2 (GCN2) in activated T cells binds to uncharged tRNAs to sense amino acid availability [29]. GCN2 activation leads to phosphorylation of eIF2 and inhibition of translation, growth impairment, and cell cycle arrest in the G_0_ phase [21].

Studies in cancer patients support an immunosuppressive role for ARG1 in evading and escaping the anti-tumor immune response. An increase in arginase activity has been observed in several cancer patients [30–33]. Increased arginase activity was detected in the peripheral blood of renal cell carcinoma patients, compared with normal controls. This activity was limited to a specific subset of myeloid cells (CD11b+, CD14-, CD15+), cells with polymorphonuclear granulocyte morphology [34]. Head and neck cancer patients also demonstrated increased ARG1 levels in immunosuppressive myeloid cells isolated from tumors, draining lymph nodes and peripheral blood [35]. In neuroblastoma, tumor cell ARG2 activity leads to both suppression of autologous and engineered anti-tumor immunity [36] . While a positive association between the levels of *ARG1* mRNA and elevated myeloid cells was observed in the peripheral blood of NSCLC patients [37], the clinical significance of these observations is currently unknown.

Based on the preclinical and clinical evidence, we evaluated the contribution of arginase mediated immunosuppression to the evasion of the anti-tumor immune responses in lung cancer. Here we first characterized the arginase expression in the primary tumors from mouse and patient lung cancers. Next, we show that in a genetically engineered mouse model (GEMM) of lung adenocarcinoma driven by KRAS^G12D^, arginase inhibition diminished growth of established tumors, which was associated with an increase in tumor T-cell infiltration and function supporting the value of arginase 1 as an immunomodulatory target for lung cancer treatment.

## Methods

### RNA sequencing of sorted immune cells

RNA sequencing data was obtained from a previously generated dataset [38]. RNA-seq reads were aligned to the mm9 Ensembl transcript annotation (release 65) using the PRADA pipeline (10.1093/bioinformatics/btu169), and FPKM expression values were determined using Cufflinks [39] with mm9 RefSeq gene annotations. FPKM values were log2-transformed and then used to calculate *p* values.

### Multiplex immunohistochemistry of TMA samples

Triple immunofluorescence (3plex IF) stains were carried in the Leica Bond-Rx fully automated staining platform (Leica Biosystems Inc., Norwell, MA). Slides were dewaxed in Bond™ Dewax solution (AR9222) and hydrated in Bond Wash solution (AR9590). Epitope retrieval for all targets were done for 30 or 20 min in Bond-epitope retrieval solution 1 pH6.0 (AR9661) or solution 2 pH9.0 (AR9640) as shown in Additional file 1 : Table S1. The epitope retrieval was followed with 10 min endogenous peroxidase blocking using Bond peroxide blocking solution (DS9800). The application order of the primary and secondary antibodies, dilutions are shown in Additional file 1: Table S1; between the stains the appropriate antigen retrieval (20 min) and peroxide blocking steps were inserted. Stained slides were counterstained with Hoechst 33258 (# H3569) and mounted with ProLong® Diamond Antifade Mountant (#P36961) Life Technologies (Carlsbad, CA). Positive and negative controls (no primary antibody) and single stain controls were done for 3plex IF when one primary antibody was omitted to make sure that cross reactivity between the antibodies did not occur.

### Reagents, media and cell lines

ID8 cells were provided by Gordon Freeman (DFCI). Cells were cultured in DMEM with 10% FBS (Heat inactivated). The rest of the cell lines were received from ATCC and cultured in their recommended media. Splenocytes were cultivated in SILAC RPMI (Thermo # 89984) media supplemented with: 10% Heat Inactivated FBS, Sodium pyruvate 1 mM, Glutamine 1 mM, Penicillin (50 U/ML) Strptomycin (50μg/ml), 2-Mercaptoethanol; 0.00005 M, (Sigma M7522), IL7: 5 ng/ml (PeproTech, #217–17 murine IL7), IL15: 100 ng/ml (PeproTech, #210–15 murine IL15), N-acetyl L Cysteine: 0.5 M (Sigma A9165), Lysine 218 uM and Arginine: 75 uM (or as indicated). Recombinant arginase was purchased from (R&D Biosystems, 5868AR010). Purified anti-mouse CD3 (cat# 100202) and purified anti-mouse CD28 (cat# 102102) antibodies were purchased from BioLegend. IFNg ELISA was performed using Biolegend (Mouse IFN-γ ELISA MAX).

### Splenocytes IFNg production assay

Splenocytes were prepared by standard techniques. Briefly, spleen was minced with syringe plunger in cell culture dish with 5 mL of splenocyte medium. The splenocytes were rinsed filtered through 40um strainer and resuspended thoroughly with 10 mL PBS + 5% FBS. Cells were spun at 400 g for 5 min. The cell pellet was resuspended in 5 mL 1X RBC Lysis Buffer and incubated at 37 C for 5 min. Lysis was stopped by adding ~ 10 mL PBS, then spinning for 5 min at 400 g. Cells were washed in 5 mL PBS, and spun for 5 min at 400 g, and resuspended in splenocyte media. IFNg secretion was stimulated by treatment with CD3 (5μg/ml) and CD28 (1μg/ml) antibodies. Sorted CD11b + myeloid cells or IL4/PGE2 treated peritoneal macrophages were added to the incubation mix when indicated. Supernatants were collected 24 h after cell culture/compound addition, and 10 to 20 ul of the sample (supernatants) was evaluated by ELISA. CD11b + myeloid cells were sorted from ID8 tumor bearing mouse ascites. Cells were isolated from ascites fluid and stained with CD45 and Cd11b antibodies. CD45+ and CD11b + cells were sorted by flow cytometry. Peritoneal macrophages were isolated by standard techniques from wild type mice after treatment with 3% thioglycolate (Sigma, T0632) for 4 days. PM were treated with IL4 (50 ng/ml) and pGE2 (Sigma P0409) (2.6 uM) for indicated times in RPMI medium (2% FBS).

### Cancer cell line IC50 assays

Compound 9 (PubChem CID**:** 66833213) was previously described by another group [40]. We purchased the compound from Wuxi LabNetwork (Shanghai, China) for in vivo studies and from MedChemExpress for in vitro studies. Compound 9 was dissolved in DMSO and used in the IC50 assays in the indicated doses. Cell titer glo (Promega) was used per the manufacturer’s instructions to determine cell viability. Cisplatin was purchased from UT Southwestern Medical Center pharmacy and diluted in saline, and stausporine was purchased from Cell Signaling Technologies.

### Detection of Arginase

Total protein lysates were prepared with NP40 lysis buffer (Boston BioProducts) with Protease inhibitors (Complete, Roche). Proteins were quantitated and 50 μg protein was resolved on SDS gel. Proteins were then transferred to PVDF membrane overnight in cold room. Blot was incubated with blocking buffer (5% milk in 1X TBST) for one hour at room temperature and then with Arginase 1 (Sigma, SAB2108087) and tubulin antibodies (Cell Signaling, #2148) overnight in cold room on a shaking platform. After washing 3 times with 1X TBST (each 10 min at room temperature), HRP conjugated secondary antibody was added to the blot for another hour at room temperature. The blot was again washed 3 times with 1X TBST, then ECL reagent was used to reveal the signals on Fluro Chem (Protein Simple). *Arg1* mRNA expression information was obtained from the Cancer Cell line Encyclopedia (CCLE) [41].

### In vivo studies and tumor immune profiling

Mice were treated with compound 9 at 30 mg/kg (dissolved in water) daily, by oral gavage. MRI was performed using the 7 Tesla Bruker MRI machine at the Lurie Family Imaging Center of DFCI. Tumor volumes were quantified using 3D Slicer software. All mouse experiments were performed with the approvals of IACUC at the Dana-Farber Cancer Institute and UT Southwestern Medical Center. Mice were of mixed 129/Sv, C57Bl/6, and BALB/c background. Immune analysis of mouse tumor tissues were performed by generating single cell suspension of tumors by collagenase treatment, lysing red blood cells, and then staining with the pertinent antibodies. Antibodies used are listed in Additional file 1: Table S2 and detailed tissue processing and staining protocol is described in Additional file 1: supplementary methods.

## Results

### Subpopulation of myeloid cells in tumors express arginase 1

To evaluate the expression of arginase in different cellular compartments in lung tumors, we sorted tumor associated macrophages (CD11c+ CD11b- CD103-) and tumor associated neutrophils (CD11b+, LY6G+) from the tumors of a classical mouse model for lung adenocarcinoma-(KRAS^G12D^ GEMM) and isolated RNA. Combination of these two populations add up to approximately 80% of all CD45+ cells in the tumor bearing lungs in our model. Lymphocytes are about 15% of all CD45+ cells, leaving only 5–7% of CD45+ to the rarer myeloid cell types. Since the majority of the myeloid cell population fall into these two major groups, we focused our analysis on these subsets (Additional file 2: Figure S1).

**Fig. 1:**
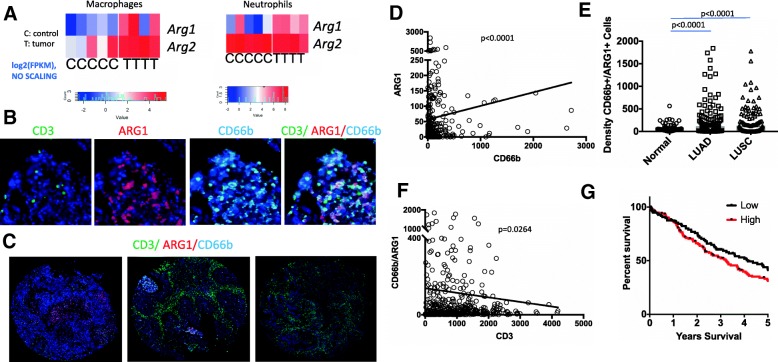
Arginase expression in the lung tumors. **a** RNA Sequencing analysis of sorted tumor (Epcam+), macrophage (CD11c+ CD11b- CD103-) and neutrophils (CD11b+, LY6G+) from Kras^G12D^ lung tumors. T denotes cells isolated from tumor carrying mice and c from healthy control littermates. Heatmaps denote Log2 of fragments per kilobase of transcript (FPKM) per each mouse sample. *p* values are 0.02 and 0.03 for macrophages and neutrophils for *Arg1* and 0.01 and 0.07 for macrophages and neutrophils for Arg2. Log2 (FPKM) values were also used for statistical analysis by student’s T test. Heatmaps represent unscaled values. **b** High power image of ARG1, CD3, CD66b multiplex immunohistochemistry. **c** Low power representative images from ARG1, CD3, CD66b multiplex immunohistochemistry staining of the TMA for varying levels of Arg1 and CD3 staining. Left: Arg1 high CD3 low, middle: Arg1 and CD3 high, and right: Arg1 low CD3 high tumor sample. **d** Correlation of CD66b and ARG1 in lung tumor samples. *P* < 0.0001, Spearman correlation. **e** Automated quantification of the ARG1 + CD66b + density in adjacent matched-normals, LUAD, and LUSQ *p* < 0.0001, determined by Mann-Whitney test **f** Correlation of CD66b+ ARG1+ double staining and CD3 staining in NSCLC, *p* < 0.0264, Spearman correlation. G. Kaplan Meier survival analysis for patients whose tumors were CD66b+/ARG1+ high or low density based on multiplex IHC. *p* = 0.09 (Log Rank test). The thresholds set for fluorescence positive signals for CD66b and CD3 are higher than that for ARG1 therefore the quantity of the expression is relative not exact

We performed RNA sequencing and validated the cell type specific markers, *Cd11b*, *Cd11c*, *Cd45* and *Epcam* [38]. Based on RNA-sequencing data, as compared to normal healthy control mice, neutrophils and macrophages from the tumors showed significantly increased expression of *Arg 1* (Fig. 1a, *p* values 0.02 and 0.03 for macrophages and neutrophils respectively). *Arg2* expression was in general higher than *Arg1* in all the sorted samples and only significantly elevated in macrophages (p values are 0.01 and 0.07 for macrophages and neutrophils).

To determine whether this is applicable to human samples, we analyzed *ARG1* and *ARG2* expression in a previously published dataset reporting single cell sequencing of patient lung tumors [42]. *ARG1* expression was only detected in myeloid cells, B cells, and some T cells. *ARG1* expression was overall very low in general across all cell types (most of the cells expressed less than < 0.004, log10 normalized), making it hard to make reliable conclusions. Arginase 2 was relatively higher and was present in most cell types within the lung microenvironment both in adjacent normal tissues and tumors (Additional file 2: Figure S2A,B).

To validate arginase 1 expression in the patient samples, we performed immunohistochemistry on formalin fixed paraffin embedded patient tumor samples in a tissue microarray (TMA) consisting of 150 lung adenocarcinoma (LUAD), 103 lung squamous cancer (LUSC), and 54 matched normal samples placed in duplicate cores. The patient cohort was representative of the general population of patients with NSCLC with median patient age of 64 years (range 41–90) for LUAD patients and 66 years (range 41–88) for LUSC patients. A high percentage of the patients were current/former smokers 73% for LUAD and 88% for LUSC (Additional file 1: Table S3.). As recently described [43], we performed multiplex immunohistochemistry for arginase1, CD66b for neutrophils, and CD3 for lymphocytes (Fig. 1b, c). Arginase expression was mainly localized in neutrophils and the arginase and CD66b stainings were significantly correlated (Fig. 1d, *p* < 0.001).

As in the mouse tumor samples, arginase expression in neutrophils was significantly elevated in patient lung tumors. CD66b + ARG1+ double positive cell densities were much higher in both LUAD and LUSC samples compared with matched normal lung samples (Fig. 1e, *p* < 0.0001). We also performed similar analysis in CXCR2+ ARG1+ stained TMA. *CXCR2* is more broadly expressed by the majority of myeloid cell groups than *CD66b/CEACAM8* (Additional file 2: Figure S2C,D^1^).

Similar to CD66b TMA, CXCR2+ ARG1+ expressing cells were elevated in lung tumors as compared to the controls (Additional file 2: Figure S3A, B). ARG1 stainings between the CD3, CD66b, ARG1 TMA and CXCR2, ARG1 TMA correlated significantly (Additional file 2: Figure S3C) highlighting technical robustness of staining and evaluation. There was also a significant correlation between densities of CXCR2 + ARG1+ and Cd66b + ARG1+ positive cells (Additional file 2: Figure S3D).

Given the role of arginase in T cell suppression we analyzed T cell presence with respect to arginase expression. There was a significantly inverse correlation between the number of CD66b and ARG1 co-stained cells and lymphocytes (CD3+) (Fig. 1f *p* = 0.0264). There were also significant inverse correlations between numbers of CD3+ cells and CD66b stained cells (*p* = 0.001) or ARG1+ stained cells (Additional file 2: Figure S4, *p* = 0.005).

Additionally, we investigated the correlation between the expression of arginase 1 and patient survival in the TMA set and observed a worse survival in tumors with higher CD66b+/ARG1+ densities, although this was not significant (Fig. 1g, *p* = 0.09). These data suggest that arginase expression can readily be assayed on patient samples and arginase expression is elevated in NSCLCs as compared to normal lung.

### Arginase inhibition by Cpd9 prevents arginase mediated immunosuppression by myeloid cells ex vivo

Arginase inhibitors have been developed as tools to evaluate the contribution of arginine deprivation mediated immune-suppression to the tumor escape from the immune response. A recently developed arginase inhibitor previously displayed therapeutic efficacy in an in vivo model of myocardial ischemia/reperfusion injury [40]. This inhibitor (Cpd9) (R)-2-amino-6-borono-2-(2-(piperidin-1-yl)ethyl) hexanoic acid, inhibited enzymatic function human arginases I and II with IC50s of 223 and 509 nM, respectively, and was active in urea release cellular assay using chicken hamster ovary (CHO) cells overexpressing human arginase I (IC50 8uM). Our characterization of Cpd9 provided similar results in enzymatic assays (human ARG1 IC50 1.1uM; human ARG2 IC50 2.7 uM) and in a HepG2 urea release assay (IC50 8 uM) (Data not shown). Given the importance of arginase activity in cancer, we further characterized this compound in ex vivo assays designed to assess the effects of arginine deprivation in T-cell responses.

**Fig. 2:**
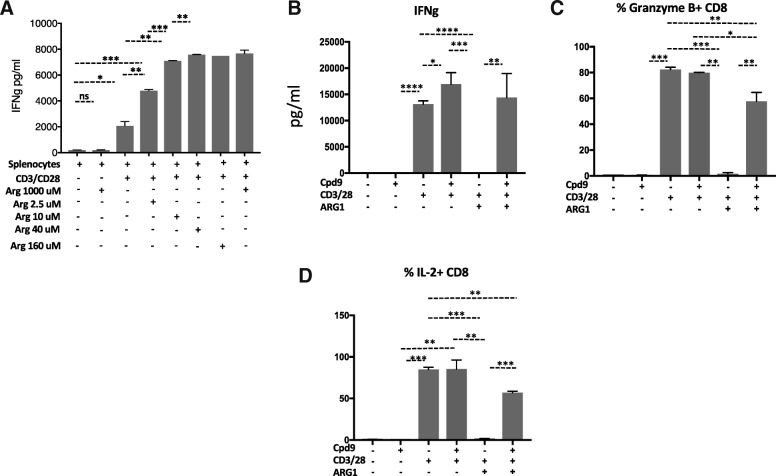
Compound 9 reverses arginine depletion mediated interferon gamma production. **a** Arginine depletion prevented IFNg secretion by splenocytes. Splenocytes were activated with CD3/28 in the presence of media with arginine levels of 0, 2.5, 10, 40, 160 and 1000 uM. After 24 h IFNg was determined by ELISA in the culture supernatants **b-d.** Compound 9 mediated restoration of T cell function. Splenocytes were isolated from mixed background mice, dissociated and activated with CD3/28 antibodies in the presence of in the presence of arginine 40 uM. Recombinant Arginase (ARG1, 1 μg/ml) and Cpd9 (50 uM) were added to the indicated wells at the same time. **b** IFNg was determined by ELISA in the culture supernatants after 24 h **c** Granzyme B expression CD8 T cells were determined by flow cytometry **d** IL-2 expressing CD8 T cells were determined by flow cytometry. *p < =0.05, **p < =0.01, and ***p < =0.001. Graphs show representative results from experiments performed at least three times

T-cell functional responses can be evaluated ex vivo by monitoring IFNγ secretion by mouse splenocytes. Arginine deprivation diminished T-cell functionality by impairing T-cell proliferation and reducing IFNγ secretion (Fig. 2a). In our studies, IFNγ secretion by mouse splenocytes was optimal when arginine levels were above 10uM, showing a significant reduction in media lacking arginine (Fig. 2a). Since the physiological concentrations of arginine are 75–150 uM [44], all IFNγ secretion assays were performed at concentrations in this range. In humans, arginine deprivation could also result from the action of secreted ARG1 to the microenvironment [45]. Addition of human ARG1 to mouse splenocytes reduced IFNγ secretion by ~ 90%, which was reversed by addition of 5–50 uM of Cpd9 (Fig. 2b and Additional file 2: Figure S5A). We have also confirmed the reversal of IL-2 and Granzyme B production with Cpd9 in these splenocytes (Fig. 2b and Additional file 2: Figure S5A).

**Fig. 3:**
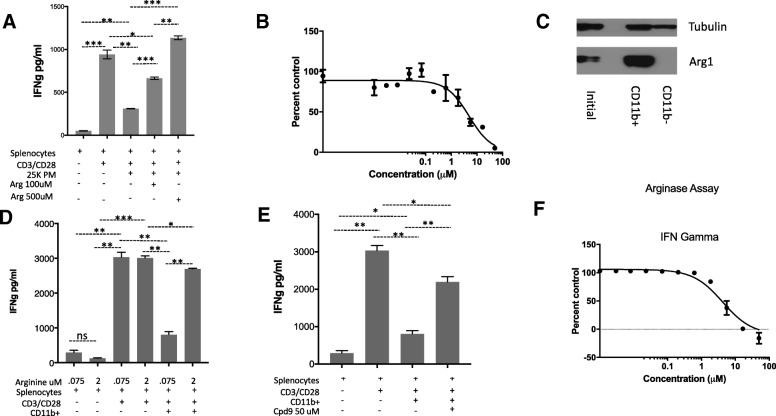
M2 macrophages and Cd11b + cells express Arginase 1 and suppress T cell function. **a** Cpd9 50 uM prevented M2 peritoneal macrophages suppression of IFNg secretion by splenocytes. Splenocytes were activated with CD3/28 in the presence of 25,000 M2 peritoneal macrophages supplemented with arginine. IFNg was determined by ELISA in the culture supernatants after 24 h. Splenocytes 50K, PM 25K. **b** IC50 of Cpd9 in 15K M2 peritoneal macrophages. **c** Western blot analysis of Arginase 1 expression in CD11b + cells. CD11b + cells were sorted from ID8 tumor ascites fluid, and Arginase 1 expression was characterized by western blot using Arginase 1 and tubulin antibodies. **d** Arginine 2 mM prevented CD11b + cells suppression of IFNg secretion by splenocytes. Splenocytes were activated with CD3/28 in the presence of 0.075 mM or 2 mM arginine as indicated. Sorted CD11b + cells were added to the indicated wells. After 24 hs IFNg was determined by ELISA in the culture supernatants. Splenocytes 50K; CD11b: 150K **e**. Cpd9 50 uM prevented CD11b + MDSC cells suppression of IFNg secretion by splenocytes. Splenocytes were activated with CD3/28 in the presence of arginine 75 uM, sorted CD11b + MDSC and Cpd9 50 uM were added to the indicated wells. IFNg was determined by ELISA in the culture supernatants after 24 h. Splenocytes 50K; CD11b: 150K **f** IC50 of Cpd9 in CD11b MDSC suppression of splenocytes IFNg secretion. Splenocytes were activated with CD3/28 in the presence of arginine 75 uM arginine, sorted CD11b + MDSC and various concentrations of Cpd9. After 24 hs IFNg was determined by ELISA in the culture supernatants and the % of control (no Cpd9) was calculated. Splenocytes 100K, Arginase 0.35 μg/ml, and Arginine 75 uM. *p < =0.05, **p < =0.01, and ***p < =0.001. Graphs show representative results from experiments performed at least three times

In both mouse and humans, arginine deprivation has been associated MDSCs expressing high levels of arginase 1 [46] . Immunosuppressive cytokines (IL-4/10) and prostaglandins (PGE2) induce the expression of arginase 1 while promoting the immunosuppressive function of myeloid cells. We treated peritoneal macrophages with IL4, IL10 and PGE2 and observed an increase in arginase 1 expression (Additional file 1: Figure S5B). The addition of these macrophages to mouse splenocytes reduced IFNg secretion by ~ 80%, which was reversed in the presence of higher concentrations of arginine in the media (Fig. 3a). Cpd9 restored IFNg with an IC50 of 4.9 uM (Fig. 3b).

In tumor bearing mice, high arginase 1 expression has been observed in myeloid cells that are Gr1 + CD11b+ [47]. Here we use the transplantable murine ID8 ovarian tumor model in wild type B6 mice, the best available transplantable tumor model associated with the recruitment of massive numbers of leukocytes including a substantial population of MDSCs [48]. This model develops ascites containing myeloid cells expressing high levels of arginase 1 [49]. We sorted CD11b + cells from ascites derived from the ID8 tumor model and confirmed high arginase 1 expression in these myeloid cells (Fig. 3c). The addition of CD11b + cells to mouse splenocytes reduced IFNγ secretion by 75%, which was completely reversed in the presence of high concentration of arginine (2 mM) (Fig. 3d). CD11b mediated suppression of IFN gamma positively correlated with the CD11b cell numbers in the co-culture condition (Additional file 2: Figure S6). Cpd9 50 uM restored IFNγ secretion with an IC50 of 8 uM consistent with its activity in previous cellular assays (Fig. 3e, f).

Arginine deprivation impaired T-cell functional responses as monitored by IFNγ secretion. The presence of ARG1 or immunosuppressive cells expressing high levels of this enzyme in the tumor microenvironment has been described as the molecular mechanism leading to arginine deprivation in the tumor microenvironment. In our assays the arginase inhibitor, Cpd9, restored IFNγ secretion in the T-cells in the presence of these two immunosuppressive conditions.

### Arginase inhibition prevents tumor growth and promotes an anti-tumor immune environment

Previous studies suggested arginase 2 expression and activity is important for breast and renal cancer cell growth in vitro by replenishing the intracellular polyamine pools and other mechanisms yet to be discovered [50, 51]. We surveyed ARG1 expression in panel of twelve human cancer cell lines from originating from different tissues to first determine whether cancer cells are dependent on arginase activity in vitro and then to determine whether tumor cell arginase expression correlates with sensitivity to Cpd9. In line with previous studies, we did not find any expression of arginase 1 across several human tumor cell lines by western blot (not shown). We detected arginase 2 in several lines by western blot (Additional file 2: Figure S7A). Compound 9 sensitivity of different human cell lines correlated with their arginase 2 expression (CCLE, Additional file 2: Figure S7B-D and data not shown).

To evaluate the cytotoxic activity of compound 9 specifically in lung cancer cell lines, we treated a panel of murine and human lung cancer cell lines with compound 9. In vitro treatment of syngeneic murine lung cancer line Lewis Lung Carcinoma (LLC), KP9–1, a cell line derived from concurrent Kras and p53 (Kras^G12D^, p53^−/−^) mutant mouse lung tumor, and human lung adenocarcinoma cancer cell lines A549 and H460 revealed no cytotoxic activity (Additional file 2: Figure S7D and data not shown).

**Fig. 4:**
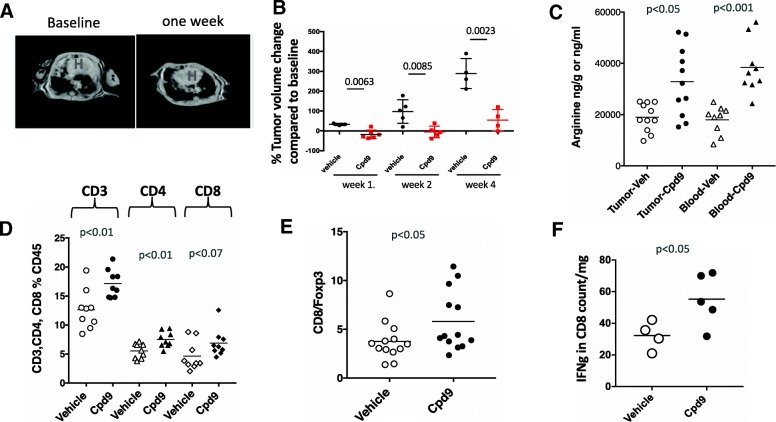
Inhibiting arginase activity has therapeutic efficacy in Kras mutant Gemms by creating an immune favorable microenvironment. **a** Representative MRI image of Kras mutant mouse lung tumors from mice treated with Compound 9 for one week. **b** Quantification of MRI images of lung tumors of mice either treated with vehicle or compound 9 for up to four weeks, each dot represents an individual mouse. *p* values for each of the 1,2 and 4 week time points are indicated on the graph **c** Measurement of arginine levels in the lung tumor tissue and blood of mice either treated with vehicle or compound 9 by metabolomics. **d** % CD3, CD4, and CD8 T cells in all CD45 cells, and **e** CD8 to regulatory T cell (FoxP3+) ratios in the lung tumor microenvironment of mice treated with vehicle or compound 9. determined by multicolor flow cytometry analysis. **f** Quantification of interferon gamma production by flow cytometry of ex vivo stimulated and enriched lymphocytes from the lung tumors in mice treated with vehicle or compound 9 for one week. All p values in this figure were determined by Student’s t test. Each data point in the graphs is from a different mouse

To evaluate the contributions of immunosuppression mechanisms driven by arginine deprivation to evasion of the anti-tumor immune response in vivo, we studied the effect of Cpd9 in an immune-competent genetically engineered mouse model of Kras driven lung adenocarcinoma. Kras is the most common oncogene detected in lung cancer patients [5]. LSL-Kras G12D transgenic mice develop sporadic pulmonary adenocarcinomas following intranasal administration of Cre-expressing adenovirus vector to activate the latent oncogene Kras G12D allele. We induced tumor formation in the LSL-Kras^G12D^ and monitored tumor growth by magnetic resonance imaging (MRI). After confirming tumor growth, we initiated treatment studies. Upon administration of 30-mg/kg of Cpd9 daily to GEMMs G12D, we detected a reduction in tumor volume as early as one week measured by MRI (Figs. 4a, b). Upon extension of this treatment up to four weeks, we detected a significant difference in the tumor growth rate as compared to baseline between the Cpd9 vs vehicle treated animals (Figs. 4a, b). Cpd9's effects on tumor growth correlated with its pharmacodynamic effects on tumor and blood arginine levels. Cpd9 treatment significantly increased the levels of arginine in both tumor (*p* < 0.05) and blood (*p* < 0.001) (Fig. 4c). These results are consistent with Arginase inhibition preventing arginine deprivation, which in turn leads to reduction in tumor size in transgenic Kras G12D mice.

To understand the impact of Cpd9 on the tumor microenvironment, we compared the lung immune cell populations by flow cytometry among animals treated with vehicle or Cpd9 30 mpk for one week. The arginine increment elicited by Cpd9 was associated with an increase in tumor T cells, the levels of CD3, CD4 and CD8 cells increased in the tumor microenvironment (CD3 *p* < 0.01, CD4 *p* < 0.007 and CD8 *p* < 0.07) (Fig. 4d). Tumors from vehicle treated samples presented lower T-cell levels than naïve or non-induced mouse controls (data not shown), which were restored upon Cpd9 treatment. This increase in T-cells was accompanied by a significant increase in the CD8/Foxp3 ratio (*p* < 0.05), and a significant increase in IFN gamma production in ex vivo culture of T cells isolated from the tumors (Fig. 4e, f). The number of myeloid population of the immune infiltrate showed no significant difference between Cpd9 or vehicle treated samples; we detected similar levels of alveolar macrophages and granulocytes in both samples (not shown). These data combined with the lack of cytotoxicity with cell line studies suggests that the in vivo effect of the Cpd 9 in Kras mouse lung tumor model is cell non-autonomous and Cpd9 exhibits antitumor effects through arginine restoration, T cell recruitment to the tumor sites, and improved T cell function.

## Discussion

Metabolic balance in the tumor microenvironment is important for T cell survival, activation, and anti-tumor functions. Arginase activity suppresses T cell function by depleting arginine from the microenvironment. Compound 9, a novel inhibitor of arginase enzymes, is able to reverse the T cell suppressive effects of arginase in ex vivo cultures and in a clinically relevant genetically engineered Kras G12D driven mouse model of lung adenocarcinoma. Furthermore, treatment with the arginase inhibitor Cpd 9 led to regression of the established tumors and delayed progression upon long-term treatment in this model.

While normal lung tissue has very little arginase expression, Arginase 1 mRNA was detected in the whole lung tissue of Kras mutant mouse [52]. Here we showed that myeloid cells in the tumors have high levels of arginase expression in both mouse and patient samples. Lower density of tumor infiltrating T cells have been associated with a poor prognosis [53], and worse response to immune checkpoint blockade treatments in patients [54, 55]. Previously, by analyzing the freshly resected surgical tumors we had shown a significant negative correlation between neutrophils and T cells in lung adenocarcinomas [56]. By staining the TMA with large numbers of patient samples, we additionally found a negative correlation between arginase expression or neutrophil counts and T cells in the tumors. This current study highlights that these types of quantitative immunological characterizations can also be done on paraffin embedded tissues.

Arginase expression has been previously shown to be induced by cyclooxgenase (COX2), prostaglandin producing enzyme in LLC lung cancer model [57]. COX2 knockout mice had reduced KRAS^G12D^ driven tumors, however COX2 had also effects in vitro tumor cell colony formation [58]. Pharmacological inhibition of COX2 led to a decrease in MDSCs and increased antitumor T cell function [57]. In a Braf driven melanoma model, COX2 inhibition synergized with immunotherapy [59]. These data suggest COX2 has roles in several aspects of tumor growth not limited to suppression of anti-tumor immunity. In this current study, we showed that peritoneal macrophages express ARG1 in response to PGE2. We specifically addressed the role of arginase enzyme function downstream of PGE2 in tumor maintenance and observed a therapeutic response in Kras driven lung cancer mouse model but not in cultured cells.

Tumor myeloid cell content is becoming to be recognized as an important marker with respect to patient outcomes. Recent data published by our group and others suggested that a higher neutrophil to lymphocyte ratio is associated with a worse prognosis in lung cancer models and patients [56, 60]. Depletion of neutrophils was shown to prevent tumor growth or [61], we have shown that it causes tumor regression [38]. Higher neutrophil to lymphocyte ratio is also is predictive of poor response to immune checkpoint blockade in both lung cancer and melanoma indicating a clinically important role for myeloid cells [62, 63]. Given the around 20% response rates to single agent checkpoint blockade [13, 64], and fewer T cells in arginase rich tumors, arginase inhibition could be investigated in the checkpoint blockade resistant tumors.

The first group of arginase inhibitors generated consisted of the boronic acid analogs of l-arginine (2)S-amino-6-hexanoic acid (ABH) and (2-boronoethyl)-l-cysteine) S-2-BEC both of which inhibit the catalytic activity of arginase. Second generation of inhibitors were based on the intermediates in L-Arginine/NO pathway: N^G^-Hydroxy-L-arginine (NOHA) and N-hydroxy-nor-l-arginine. Both NOHA and Nor-NOHA inhibit arginase activity; with nor-NOHA being a more potent inhibitor (Ki = 500 mM for nor-NOHA vs. Ki = 10 μM for NOHA) and with both being less specific BEC and ABH (reviewed in) [65]). As for Compound 9: it is 6-fold more potent relative to ABH [40]. Activity of compound 9 is similar to Nor-NOHA, however compound Cpd 9 is superior due to its specificity. Previously described arginase inhibitor Nor-NOHA was shown to delay the tumor growth of LLC model in wild type mice but not in SCID mice when injected at the tumor site at very high doses [66]. In a more recent study an arginase inhibitor was shown to delay tumor growth in syngeneic tumor models [67]. In our studies, we showed that Cpd 9 has therapeutic efficacy on established orthotopic tumors, when given systemically even at lower doses in the clinically relevant mouse lung cancer model.

Myeloid Arginase 1 was shown to be responsible for arginine depletion related immune suppression in cancer. Previous studies found no ARG1 expression in LLC tumor cell in vitro, or from the cyto-spins, and most of the expression was in the myeloid cells [66]. Another study done in GEMMs by Caetano et al., found *Arg1* in the whole lungs from the tumor bearing mice. Same study found that IL-6 blockade reduced *Arg1* expression in sorted CD11b + Ly6G- cells [52]. Single cell RNA-Seq of patient lung tumors showed very little *ARG1* in general and was elevated mainly in B cells, myeloid cells or minor group of T cells in the tumor, while *ARG2* was expressed across several cell types both in normal tissue and tumors [42]. ARG2 was shown to be important in T cell suppression in leukemia and neuroblastoma [36, 68]. In contrast, ARG2 function was shown to be not critical in lung cancer in a prior study. This study showed that intracellular ARG2 does release urea into the media but is not sufficient to deplete extracellular arginine to suppress T cell function [26]. Given that arginase 2 mRNA was expressed in the healthy tissue and not specifically elevated in the tumor both in our mice and patient tumors, we focused on characterizing arginase 1 expression in CD66b or CXCR2 expressing myeloid cells in the patient tissues. Though, the role of arginase 2 in maintaining T cell tolerance in health and disease is an area for future investigation.

## Conclusions

We show that arginase activity is elevated in in mouse and patient lung tumors and this activity inhibits T cell function. Arginase inhibition causes tumor regression by restoring arginine levels and improving T cell function in an arginase expressing mouse autochthonous tumor model.

Given that the majority of tumors are resistant to checkpoint blockade immunotherapy [69], it will be worthwhile investigating arginase activity and arginase inhibition as an immunotherapy agent in other cancer types where myeloid cells are the dominant immune cell type. Arginase expression can easily be detected by immunohistochemistry making arginase positivity a readily applicable biomarker for treatment with arginase inhibitors.

## Additional file

Additional file 1: **Table S1.** Multiplex Immunohistochemisty details for CXCR2/CD3/ARG1 and CD66b/CD3/ARG1 stainings. **Table S2.** Flow antibody list. **Table S3.** Summary of TMA Patient demographics. Supplementary methods. (PDF 97 kb)

Additional file 2: **Figure S1.** Profiling of immune cells in mouse lung tumor microenvironment: Top: Gating strategy for alveolar macrophages, neutrophils, and other myeloid cells. Bottom left: Lung weights in mg. Bottom right:Quantification of Alveolar macrophages, neutrophils, lymphocytes, and other rare types. **Figure S2.** Expression of myeloid markers and ARG1 and ARG2 in all cell types in the lung tumor microenvironment in published dataset. **Figure S3.** Analysis of staining for myeloid marker CXCR2 and ARG1 in NSCLC TMA. **Figure S4.** Correlation of Cd66b, and Arginase, and CD3 in multiplex IHC stained Tissue microarray. **Figure S5.** Evaluation of the activity of Cpd 9 at different doses and Arg1 expression in peritoneal macrophages. **Figure S6:** CD11b + MDSCs sorted from ID8 tumor ascites reduced of IFNg secretion by splenocytes. **Figure S7.** Arginase inhibitor sensitivity correlates with Arginase expression in cancer cell lines. (PDF 1331 kb)

## References

[1] Siegel RL, Miller KD, Jemal A (2017). Cancer statistics, 2017. CA Cancer J Clin.

[2] Friboulet L, Li N, Katayama R, Lee CC, Gainor JF, Crystal AS (2014). The ALK inhibitor ceritinib overcomes crizotinib resistance in non-small cell lung cancer. Cancer Discov.

[3] Shaw AT, Kim DW, Nakagawa K, Seto T, Crino L, Ahn MJ (2013). Crizotinib versus chemotherapy in advanced ALK-positive lung cancer. N Engl J Med.

[4] Lynch TJ, Bell DW, Sordella R, Gurubhagavatula S, Okimoto RA, Brannigan BW (2004). Activating mutations in the epidermal growth factor receptor underlying responsiveness of non-small-cell lung cancer to gefitinib. N Engl J Med.

[5] Cancer Genome Atlas Research N (2014). Comprehensive molecular profiling of lung adenocarcinoma. Nature.

[6] Hanahan D, Weinberg RA (2011). Hallmarks of cancer: the next generation. Cell.

[7] Drake CG, Jaffee E, Pardoll DM (2006). Mechanisms of immune evasion by tumors. Adv Immunol.

[8] Whatcott C, Han H, Posner RG, Von Hoff DD (2013). Tumor-stromal interactions in pancreatic cancer. Crit Rev Oncog.

[9] Pardoll DM (2012). The blockade of immune checkpoints in cancer immunotherapy. Nat Rev Cancer.

[10] Postow MA, Harding J, Wolchok JD (2012). Targeting immune checkpoints: releasing the restraints on anti-tumor immunity for patients with melanoma. Cancer J.

[11] Ho PC, Liu PS (2016). Metabolic communication in tumors: a new layer of immunoregulation for immune evasion. J Immunother Cancer..

[12] Topalian SL, Hodi FS, Brahmer JR, Gettinger SN, Smith DC, McDermott DF (2012). Safety, activity, and immune correlates of anti-PD-1 antibody in cancer. N Engl J Med.

[13] Gettinger SN, Horn L, Gandhi L, Spigel DR, Antonia SJ, Rizvi NA (2015). Overall survival and long-term safety of Nivolumab (anti-programmed death 1 antibody, BMS-936558, ONO-4538) in patients with previously treated advanced non-small-cell lung Cancer. J Clin Oncol.

[14] Morgensztern D, Herbst RS (2016). Nivolumab and Pembrolizumab for non-small cell lung Cancer. Clin Cancer Res.

[15] Herbst RS, Baas P, Kim DW, Felip E, Perez-Gracia JL, Han JY (2016). Pembrolizumab versus docetaxel for previously treated, PD-L1-positive, advanced non-small-cell lung cancer (KEYNOTE-010): a randomised controlled trial. Lancet.

[16] Reck M, Rodriguez-Abreu D, Robinson AG, Hui R, Csoszi T, Fulop A (2016). Pembrolizumab versus chemotherapy for PD-L1-positive non-small-cell lung Cancer. N Engl J Med.

[17] Kareva I, Hahnfeldt P (2013). The emerging "hallmarks" of metabolic reprogramming and immune evasion: distinct or linked?. Cancer Res.

[18] Moffett JR, Namboodiri MA (2003). Tryptophan and the immune response. Immunol Cell Biol.

[19] Smith C, Chang MY, Parker KH, Beury DW, DuHadaway JB, Flick HE (2012). IDO is a nodal pathogenic driver of lung cancer and metastasis development. Cancer Discov..

[20] Sheridan C (2015). IDO inhibitors move center stage in immuno-oncology. Nat Biotechnol.

[21] Rodriguez PC, Quiceno DG, Ochoa AC (2007). L-arginine availability regulates T-lymphocyte cell-cycle progression. Blood.

[22] Ensor CM, Holtsberg FW, Bomalaski JS, Clark MA (2002). Pegylated arginine deiminase (ADI-SS PEG20,000 mw) inhibits human melanomas and hepatocellular carcinomas in vitro and in vivo. Cancer Res.

[23] Morris SM (2006). Arginine: beyond protein. Am J Clin Nutr.

[24] Yang Z, Ming XF (2014). Functions of arginase isoforms in macrophage inflammatory responses: impact on cardiovascular diseases and metabolic disorders. Front Immunol.

[25] Munder M (2009). Arginase: an emerging key player in the mammalian immune system. Br J Pharmacol.

[26] Rotondo R, Mastracci L, Piazza T, Barisione G, Fabbi M, Cassanello M (2008). Arginase 2 is expressed by human lung cancer, but it neither induces immune suppression, nor affects disease progression. Int J Cancer.

[27] Rodriguez PC, Ochoa AC, Al-Khami AA (2017). Arginine metabolism in myeloid cells shapes innate and adaptive immunity. Front Immunol.

[28] Corzo CA, Condamine T, Lu L, Cotter MJ, Youn JI, Cheng P (2010). HIF-1alpha regulates function and differentiation of myeloid-derived suppressor cells in the tumor microenvironment. J Exp Med.

[29] Dong J, Qiu H, Garcia-Barrio M, Anderson J, Hinnebusch AG (2000). Uncharged tRNA activates GCN2 by displacing the protein kinase moiety from a bipartite tRNA-binding domain. Mol Cell.

[30] Wu CW, Chi CW, Tsay SH, Lui WY, P’Eng FK, Chang KL (1987). The effects of arginase on neoplasm. I. The role of arginase in the immunosuppressive effects of extract from gastric cancer. Zhonghua Min Guo Wei Sheng Wu Ji Mian Yi Xue Za Zhi.

[31] Wu CW, Chi CW, Lin EC, Lui WY, P’ Eng FK, Wang SR (1994). Serum arginase level in patients with gastric cancer. J Clin Gastroenterol.

[32] Polat MF, Taysi S, Polat S, Boyuk A, Bakan E (2003). Elevated serum arginase activity levels in patients with breast cancer. Surg Today.

[33] Porembska Z, Luboinski G, Chrzanowska A, Mielczarek M, Magnuska J, Baranczyk-Kuzma A (2003). Arginase in patients with breast cancer. Clin Chim Acta.

[34] Ochoa AC, Zea AH, Hernandez C, Rodriguez PC. Arginase, prostaglandins, and myeloid-derived suppressor cells in renal cell carcinoma. Clin Cancer Res 2007;13(2 Pt 2):721s–726s. 10.1158/1078-0432.CCR-06-2197 17255300

[35] Vasquez-Dunddel D, Pan F, Zeng Q, Gorbounov M, Albesiano E, Fu J (2013). STAT3 regulates arginase-I in myeloid-derived suppressor cells from cancer patients. J Clin Invest.

[36] Mussai F, Egan S, Hunter S, Webber H, Fisher J, Wheat R (2015). Neuroblastoma arginase activity creates an immunosuppressive microenvironment that impairs autologous and engineered immunity. Cancer Res.

[37] Heuvers ME, Muskens F, Bezemer K, Lambers M, Dingemans AM, Groen HJ (2013). Arginase-1 mRNA expression correlates with myeloid-derived suppressor cell levels in peripheral blood of NSCLC patients. Lung Cancer.

[38] Koyama S, Akbay EA, Li YY, Aref AR, Skoulidis F, Herter-Sprie GS (2016). STK11/LKB1 deficiency promotes neutrophil recruitment and Proinflammatory cytokine production to suppress T-cell activity in the lung tumor microenvironment. Cancer Res.

[39] Trapnell C, Williams BA, Pertea G, Mortazavi A, Kwan G, van Baren MJ (2010). Transcript assembly and quantification by RNA-Seq reveals unannotated transcripts and isoform switching during cell differentiation. Nat Biotechnol.

[40] Van Zandt MC, Whitehouse DL, Golebiowski A, Ji MK, Zhang M, Beckett RP (2013). Discovery of (R)-2-amino-6-borono-2-(2-(piperidin-1-yl)ethyl)hexanoic acid and congeners as highly potent inhibitors of human arginases I and II for treatment of myocardial reperfusion injury. J Med Chem.

[41] Barretina J, Caponigro G, Stransky N, Venkatesan K, Margolin AA, Kim S (2012). The Cancer cell line encyclopedia enables predictive modelling of anticancer drug sensitivity. Nature.

[42] Lambrechts D, Wauters E, Boeckx B, Aibar S, Nittner D, Burton O (2018). Phenotype molding of stromal cells in the lung tumor microenvironment. Nat Med.

[43] Porrello A, Leslie PL, Harrison EB, Gorentla BK, Kattula S, Ghosh SK (2018). Factor XIIIA-expressing inflammatory monocytes promote lung squamous cancer through fibrin cross-linking. Nat Commun.

[44] Vissers YL, Dejong CH, Luiking YC, Fearon KC, von Meyenfeldt MF, Deutz NE (2005). Plasma arginine concentrations are reduced in cancer patients: evidence for arginine deficiency?. Am J Clin Nutr.

[45] Pallett LJ, Gill US, Quaglia A, Sinclair LV, Jover-Cobos M, Schurich A (2015). Metabolic regulation of hepatitis B immunopathology by myeloid-derived suppressor cells. Nat Med.

[46] Wesolowski R, Markowitz J, Carson WE (2013). Myeloid derived suppressor cells - a new therapeutic target in the treatment of cancer. J Immunother Cancer..

[47] Youn JI, Nagaraj S, Collazo M, Gabrilovich DI (2008). Subsets of myeloid-derived suppressor cells in tumor-bearing mice. J Immunol.

[48] Hart KM, Byrne KT, Molloy MJ, Usherwood EM, Berwin B (2011). IL-10 immunomodulation of myeloid cells regulates a murine model of ovarian cancer. Front Immunol.

[49] Bak SP, Alonso A, Turk MJ, Berwin B (2008). Murine ovarian cancer vascular leukocytes require arginase-1 activity for T cell suppression. Mol Immunol.

[50] Tate DJ, Jr., Vonderhaar DJ, Caldas YA, Metoyer T, Patterson JRt, Aviles DH, et al. Effect of arginase II on L-arginine depletion and cell growth in murine cell lines of renal cell carcinoma. J Hematol Oncol 2008;1:14. 10.1186/1756-8722-1-14 PMC2562378 18817562

[51] Singh R, Pervin S, Wu G, Chaudhuri G (2001). Activation of caspase-3 activity and apoptosis in MDA-MB-468 cells by N(omega)-hydroxy-L-arginine, an inhibitor of arginase, is not solely dependent on reduction in intracellular polyamines. Carcinogenesis.

[52] Caetano MS, Zhang H, Cumpian AM, Gong L, Unver N, Ostrin EJ (2016). IL6 blockade reprograms the lung tumor microenvironment to limit the development and progression of K-ras-mutant lung Cancer. Cancer Res.

[53] Skoulidis F, Byers LA, Diao L, Papadimitrakopoulou VA, Tong P, Izzo J (2015). Co-occurring genomic alterations define major subsets of KRAS-mutant lung adenocarcinoma with distinct biology, immune profiles, and therapeutic vulnerabilities. Cancer Discov..

[54] Tumeh PC, Harview CL, Yearley JH, Shintaku IP, Taylor EJ, Robert L (2014). PD-1 blockade induces responses by inhibiting adaptive immune resistance. Nature.

[55] Ji RR, Chasalow SD, Wang L, Hamid O, Schmidt H, Cogswell J (2012). An immune-active tumor microenvironment favors clinical response to ipilimumab. Cancer Immunol Immunother.

[56] Akbay EA, Koyama S, Liu Y, Dries R, Bufe LE, Silkes M, et al. Interleukin-17A promotes lung tumor progression through neutrophil attraction to tumor sites and mediating resistance to PD-1 blockade. J Thorac Oncol. 2017. 10.1016/j.jtho.2017.04.017 PMC5532066 28483607

[57] Rodriguez PC, Hernandez CP, Quiceno D, Dubinett SM, Zabaleta J, Ochoa JB (2005). Arginase I in myeloid suppressor cells is induced by COX-2 in lung carcinoma. J Exp Med.

[58] Pan Y, Jiang Y, Tan L, Ravoori MK, Gagea M, Kundra V (2015). Deletion of cyclooxygenase-2 inhibits K-ras-induced lung carcinogenesis. Oncotarget.

[59] Zelenay S, van der Veen AG, Bottcher JP, Snelgrove KJ, Rogers N, Acton SE (2015). Cyclooxygenase-dependent tumor growth through evasion of immunity. Cell.

[60] Derman BA, Macklis JN, Azeem MS, Sayidine S, Basu S, Batus M (2017). Relationships between longitudinal neutrophil to lymphocyte ratios, body weight changes, and overall survival in patients with non-small cell lung cancer. BMC Cancer.

[61] Chang SH, Mirabolfathinejad SG, Katta H, Cumpian AM, Gong L, Caetano MS (2014). T helper 17 cells play a critical pathogenic role in lung cancer. Proc Natl Acad Sci U S A.

[62] Bagley SJ, Kothari S, Aggarwal C, Bauml JM, Alley EW, Evans TL (2017). Pretreatment neutrophil-to-lymphocyte ratio as a marker of outcomes in nivolumab-treated patients with advanced non-small-cell lung cancer. Lung Cancer.

[63] Cassidy MR, Wolchok RE, Zheng J, Panageas KS, Wolchok JD, Coit D (2017). Neutrophil to lymphocyte ratio is associated with outcome during Ipilimumab treatment. EBioMedicine.

[64] Weber JS, D'Angelo SP, Minor D, Hodi FS, Gutzmer R, Neyns B (2015). Nivolumab versus chemotherapy in patients with advanced melanoma who progressed after anti-CTLA-4 treatment (CheckMate 037): a randomised, controlled, open-label, phase 3 trial. Lancet Oncol.

[65] Steppan J, Nyhan D, Berkowitz DE (2013). Development of novel arginase inhibitors for therapy of endothelial dysfunction. Front Immunol.

[66] Rodriguez PC, Quiceno DG, Zabaleta J, Ortiz B, Zea AH, Piazuelo MB (2004). Arginase I production in the tumor microenvironment by mature myeloid cells inhibits T-cell receptor expression and antigen-specific T-cell responses. Cancer Res.

[67] Steggerda SM, Bennett MK, Chen J, Emberley E, Huang T, Janes JR (2017). Inhibition of arginase by CB-1158 blocks myeloid cell-mediated immune suppression in the tumor microenvironment. J Immunother Cancer..

[68] Mussai F, De Santo C, Abu-Dayyeh I, Booth S, Quek L, McEwen-Smith RM (2013). Acute myeloid leukemia creates an arginase-dependent immunosuppressive microenvironment. Blood.

[69] Gong J, Chehrazi-Raffle A, Reddi S, Salgia R (2018). Development of PD-1 and PD-L1 inhibitors as a form of cancer immunotherapy: a comprehensive review of registration trials and future considerations. J Immunother Cancer.

