# Supplementary material for: Suppression of Myeloid Cell Arginase Activity leads to Therapeutic Response in a NSCLC Mouse Model by Activating Anti-Tumor Immunity
Source: J Immunother Cancer. 2019 Feb 6;7:32. doi: 10.1186/s40425-019-0504-5 (PMC6366094; doi:10.1186/s40425-019-0504-5)
Supplement: Supplementary file 1 — Table S1. Multiplex Immunohistochemisty details for CXCR2/CD3/ARG1 and CD66b/CD3/ARG1 stainings. Table S2. Flow antibody list. Table S3. Summary of TMA Patient demographics. Supplementary methods. (PDF 97 kb) [file 40425_2019_504_MOESM1_ESM.pdf]

Additional file 1: Supplementary methods and tables

Supplementary Table 1. Multiplex Immunohistochemisty details for CXCR2/CD3/Arg1 and CD66b/CD3/Arg1 stainings

Staining sequence	1	2	3
Antibody (clone)	**Arginase 1 (D4E3M)**	**CD66b (80H3)**	**CD3 (LN10)**
Host, Clonality M=monocl,P=polycl	Rabbit M IgG	Mouse M IgG1	Mouse M IgG2a
Vendor (Catalog #)	Cell Signaling (#93668)	LifeSpan BioSciences (#LS-B7134)	Leica (#PA0553)
Leica Antigen Retrieval (min)	Bond-epitope retrieval solution 1, pH6.0 #AR9661 (30)	Bond-epitope retrieval solution 1, pH6.0 #AR9661 (20)	Bond-epitope retrieval solution 2, p9.0 #AR9640 (20)
Peroxidase Block	10	10	10
Protein Block (min) DAKO # X0909	10		
1°Ab Dilution (1:xx)	100	100	RTU
1° Ab Inc. time	2h	3h	2h
2° Antibody	Novocastra Novolink Polymer #RE7200-K (Leica)	Novocastra Novolink Post Primary & Polymer #RE7200-K (Leica)	Novocastra Novolink Post Primary & Polymer #RE7200-K (Leica)
TSA Reagent	TSA-Cy5 #SAT705A001EA5	TSA-Cy3 #SAT704A001EA, Perkin Elmer	Alexa Fluor™ 488 Tyramide Reagent, # B40953, Life Tecnologies
Pseudo-color	Red	Cyan	Green
Detection Kit (Leica)	Bond Research Detection Kit #DS9455	Bond Research Detection Kit #DS9455	Bond Research Detection Kit #DS9455

Supplementary Table 1. continued

Staining sequence	1	2	3
Antibody (clone)	**Arginase 1 (D4E3M)**	**CXCR2 (48311)**	**CD3 (LN10)**
Host, Clonality M=monocl,P=polycl	Rabbit M IgG	Mouse M IgG2A	Mouse M IgG2a
Vendor (Catalog #)	Cell Signaling (#93668)	R&D Systems (#MAB331)	Leica (#PA0553)
Leica Antigen Retrieval (min)	Bond-epitope retrieval solution 1, pH6.0 #AR9661 (30)	Bond-epitope retrieval solution 1, pH6.0 #AR9661 (20)	Bond-epitope retrieval solution 2, p9.0 #AR9640 (20)
Peroxidase Block	10	10	10
Protein Block (min) DAKO # X0909	10		
1°Ab Dilution (1:xx)	100	50	RTU
1° Ab Inc. time	2h	3h	2h
2° Antibody	Novocastra Novolink Polymer #RE7200-K (Leica)	Novocastra Novolink Post Primary & Polymer #RE7200-K (Leica)	Novocastra Novolink Post Primary & Polymer #RE7200-K (Leica)
TSA Reagent	TSA-Cy5 #SAT705A001EA5	TSA-Cy3 #SAT704A001EA, Perkin Elmer	Alexa Fluor™ 488 Tyramide Reagent, # B40953, Life Tecnologies
Pseudo-color	Red	Cyan	Green
Detection Kit (Leica)	Bond Research Detection Kit #DS9455	Bond Research Detection Kit #DS9455	Bond Research Detection Kit #DS9455

Supplementary Table 2. Flow antibody list:

Antigen	Clone	Antigen	Clone
**CD45**	30-F11	CD44	IM7
**CD3ε**	145-2C11	PD-1	29F.1A12
**CD4**	RM4-5	TIM-3	RMT3-23
**CD8a**	53-6.7	LAG-3	C9B7W
**CD19**	6D5	BTLA	8F4
**DX5**	DX5	VISTA	MH5A
**NKp46**	29A1.4	PD-L1	10F.9G2
**CD11c**	N418	CEACAM1	CC1
**CD11b**	M1/70	FOXP3	FJK-16s
**Ly6G**	1A8	CTLA-4	UC10-4B9
**IL-2**	JES6-5H4	Granzyme B	QA16A02
**CD103**	2E7	IFNγ	XMG1.2

Supplementary Table 3: Summary of TMA Patient demographics:

	Adeno	Squamous
**Age at surgery**		
**Median**	64	66
**Range**	41-90	41-88
**Unknown**	10	0
**Gender**		
**Female**	78 (52 %)	38 (37 %)
**male**	62 (41 %)	65 (63 %)
**Unknown**	10 (7 %)	0
**Smoking status**		
**Never smoker**	26 (17 %)	
**Past or current smoker**	110 (73 %)	91 (88 %)
**Unknown**	14 (9 %)	12 (12 %)
**Pathology stage**		
**IA**	43 (29 %)	25 (24 %)
**IB**	27 (18 %)	35 (34 %)
**IIA**	9 (6 %)	6 (6 %)
**IIB**	26 (17 %)	24 (23 %)
**IIIA**	17 (11 %)	11 (11 %)
**IIIB**	2 (1 %)	1 (1 %)
**IV**	7 (5 %)	1 (1 %)
**Unknown**	19 (13 %)	0

SUPPLEMENTARY METHODS:

Analysis of single cell RNA sequencing data from lung cancer patients:

Supplementary expression data from Lambrechts et al was retrieved (1) and Log10 normalized (FPKM) values were plotted.

Mouse tumor Immune profiling:

Processing of lung tissue, staining, and flow cytometry analysis were performed as previously described (2). Mice were euthanatized and lungs were perfused with 5mM EDTA. Lung lobes were minced and incubated in dissociation buffer (100 units/ml of collagenase type IV) (Invitrogen), 10 µg/ml of DNase I (Roche), and 10% FBS in RPMI1640 medium for 45 minutes at 37 degrees. After tissue dissociation, red blood cells (RBC) were lysed with RBC lysis buffer (Gibco-10492), and cell suspensions were filtered through 70 micron cell strainer. Single cell suspension was centrifuged, and the cell pellet was dissolved in 2% FCS in HBSS, stained with Live/dead stain (Life Technologies, #L34959) and then with antibodies at 1:50 dilution. For intracellular cytokine staining, cells from whole lungs were fractionated over Ficoll-Paque (GE Healthcare) per the manufacturer's instructions. Isolated mononuclear cells were stimulated with 50 ng/ml PMA (Sigma) and 500 ng/ml Ionomycin (Sigma) for 6 hours in the presence of Golgi plug (BD Biosciences). Fixation/permeabilization buffer (eBioscience) were used for intracellular staining. Flow data acquisition was performed on a BD Canto II flow cytometer.

REFERENCES:

1. Lambrechts D, Wauters E, Boeckx B, Aibar S, Nittner D, Burton O, et al. Phenotype molding of stromal cells in the lung tumor microenvironment. Nat Med. 2018;24(8):1277-89.

2. Akbay EA, Koyama S, Carretero J, Altabef A, Tchaicha JH, Christensen CL, et al. Activation of the PD-1 pathway contributes to immune escape in EGFR-driven lung tumors. Cancer Discov. 2013;3(12):1355-63.
