# Supplementary material for: Suppression of Myeloid Cell Arginase Activity leads to Therapeutic Response in a NSCLC Mouse Model by Activating Anti-Tumor Immunity
Source: J Immunother Cancer. 2019 Feb 6;7:32. doi: 10.1186/s40425-019-0504-5 (PMC6366094; doi:10.1186/s40425-019-0504-5)
Supplement: Supplementary file 2 — Figure S1. Profiling of immune cells in mouse lung tumor microenvironment: Top: Gating strategy for alveolar macrophages, neutrophils, and other myeloid cells. Bottom left: Lung weights in mg. Bottom right:Quantification of Alveolar macrophages, neutrophils, lymphocytes, and other rare types. Figure S2. Expression of myeloid markers and ARG1 and ARG2 in all cell types in the lung tumor microenvironment in published dataset. Figure S3. Analysis of staining for myeloid marker CXCR2 and ARG1 in NSCLC TMA. Figure S4. Correlation of Cd66b, and Arginase, and CD3 in multiplex IHC stained Tissue microarray. Figure S5. Evaluation of the activity of Cpd 9 at different doses and Arg1 expression in peritoneal macrophages. Figure S6: CD11b + MDSCs sorted from ID8 tumor ascites reduced of IFNg secretion by splenocytes. Figure S7. Arginase inhibitor sensitivity correlates with Arginase expression in cancer cell lines. (PDF 1331 kb) [file 40425_2019_504_MOESM2_ESM.pdf]

Additional file 2: Supplementary figures

Supplementary Figure 1:

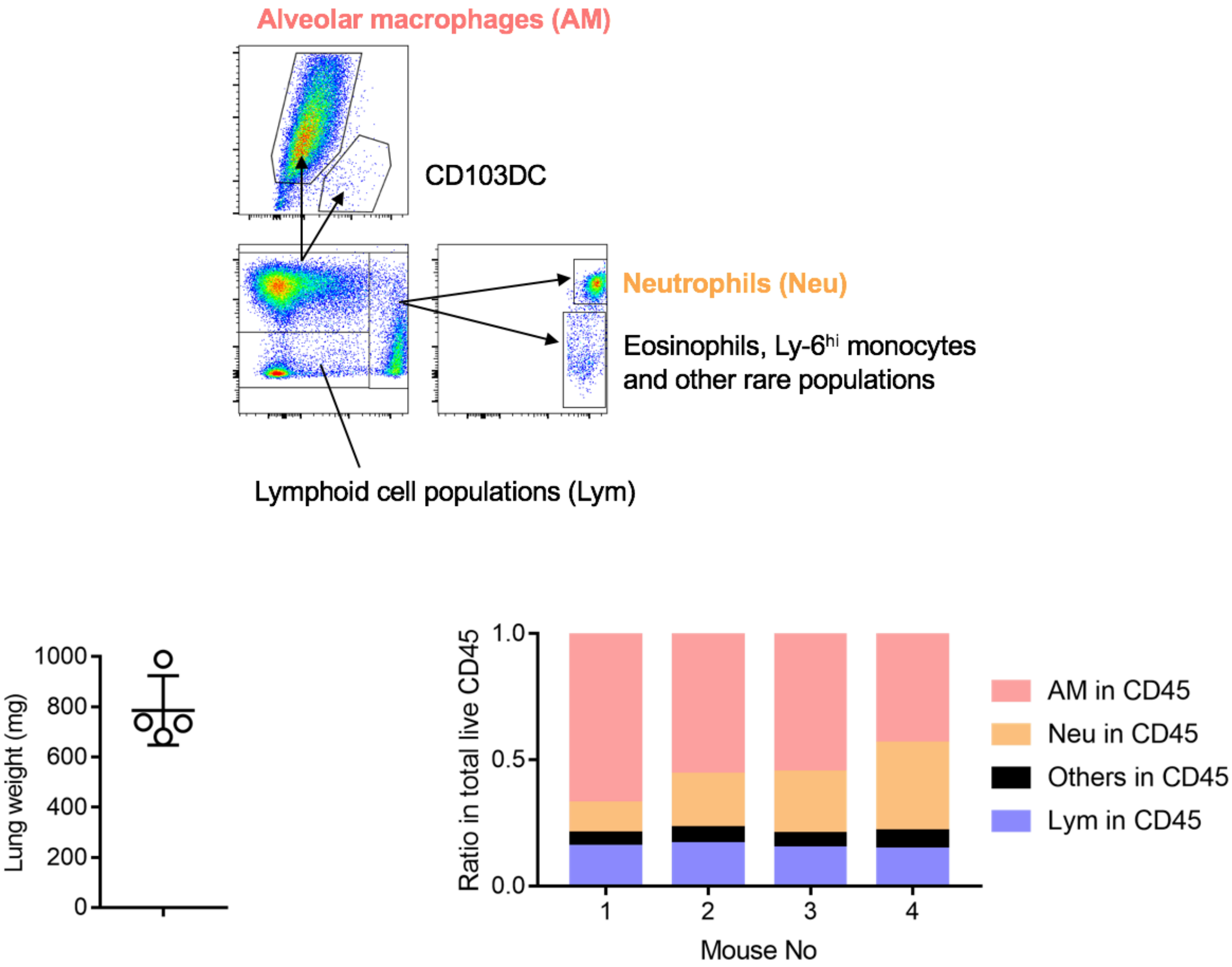

Supplementary Figure 1:

Profiling of immune cells in mouse lung tumor microenvironment: Top: Gating strategy for alveolar macrophages, neutrophils, and other myeloid cells. Bottom left: Lung weights in mg. Bottom right: Quantification of Alveolar macrophages, neutrophils, lymphocytes, and other rare types.

Supplementary Figure 2:

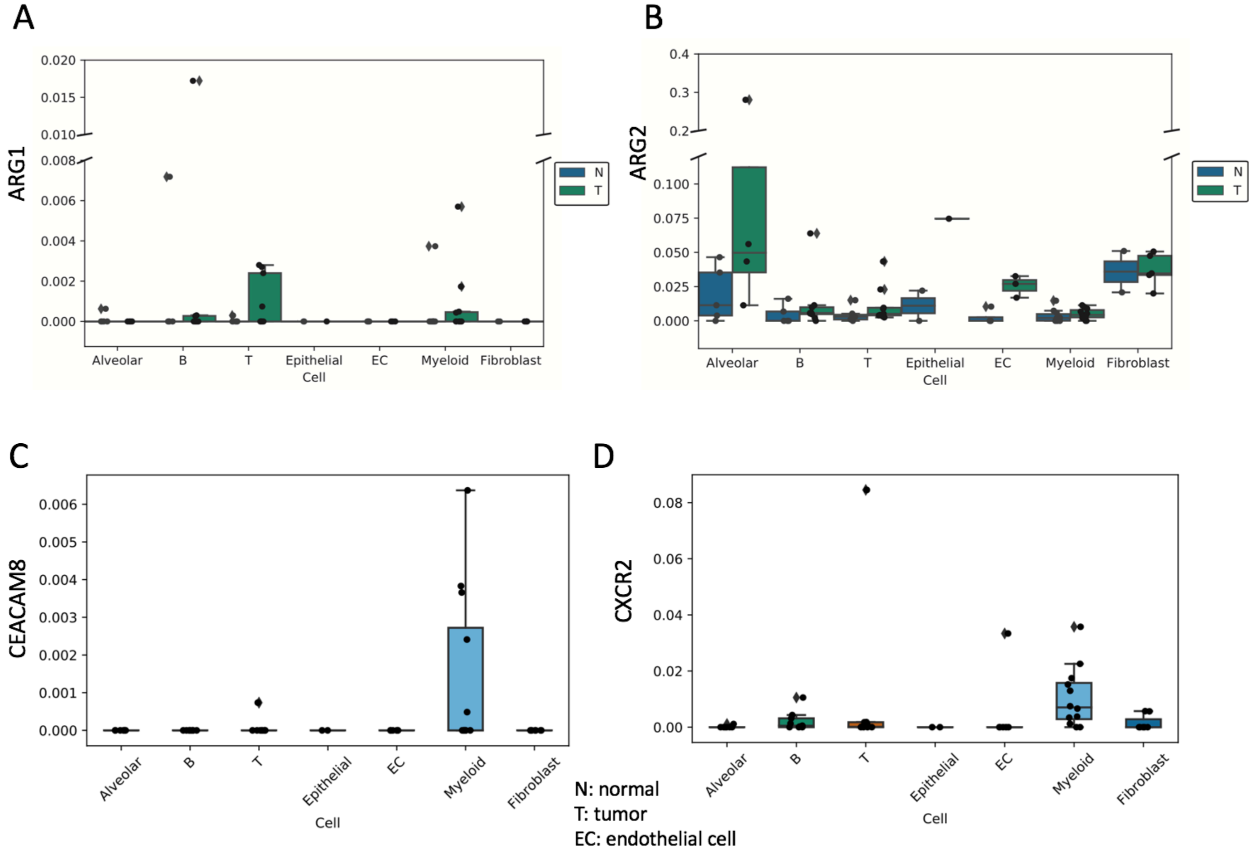

Supplementary Figure 2:
Expression of myeloid markers and ARG1 and ARG2 in all cell types in the lung tumor microenvironment in published dataset

A. CEACAM8 (CD66b) and **B.** CXCR2 expression in all the single cells from the lung tumors. Each data-point shows average per individual subtypes from the indicated group. Subtypes were determined based on expression profiling. **C.** ARG1 and **D**. Arg2 expression in normal vs lung tumors in the single cell sorted patient lung tumors and adjacent normal controls. Arg1 or Arg2 expression is not significantly different for any cell type. Graphs show log 10 normalized mRNA expression from Lambrechst et al, 2018 (see supplementary methods for the reference).

Supplementary Figure 3:

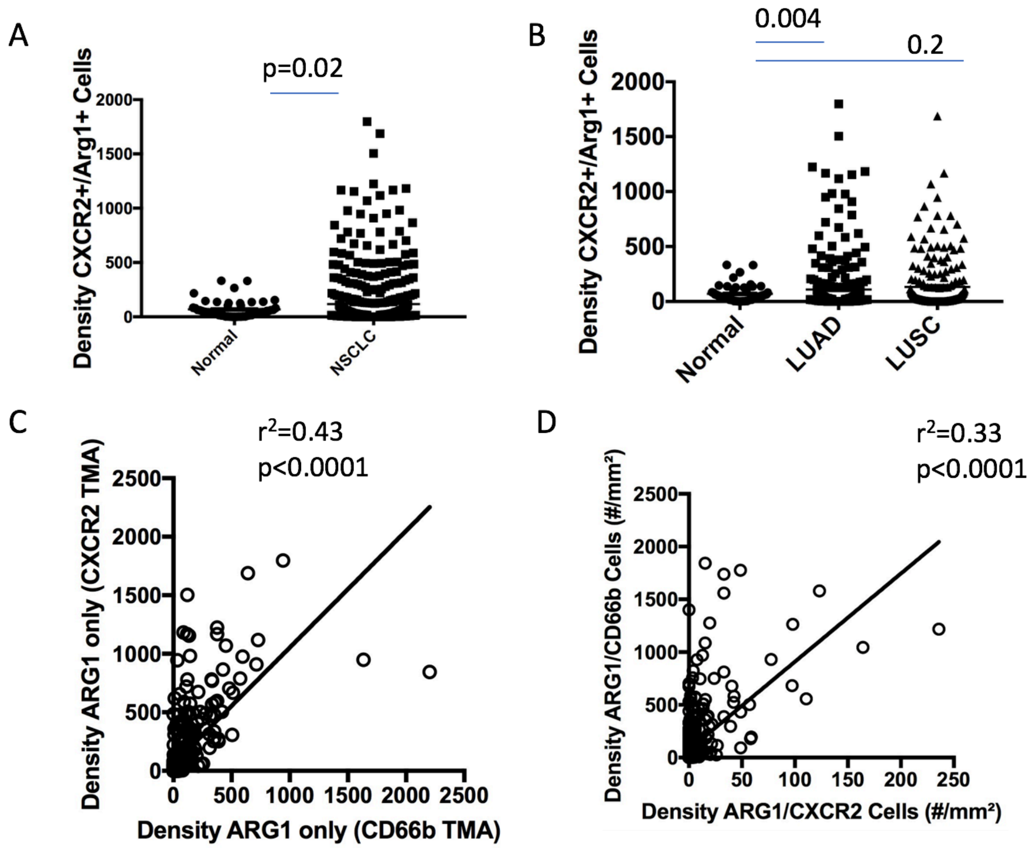

Supplementary Figure 3:

Analysis of staining for myeloid marker CXCR2 and ARG1 in NSCLC TMA

A. Automated quantification of the CXCR2+ARG1+ density in adjacent matched-normals and NSCLC p=0.02, determined by Mann-Whitney test **B.** Quantification of the CXCR2+ARG1+ density in adjacent matched-normals or LUAD or LUSQ p=0.004, and 0.2 respectively determined by Mann-Whitney test. **C.** Correlation of ARG1 staining in CXCR2+ ARG1+ and CD66b+ ARG1+ TMA (figure 1) for quality control. p<0.0001, Spearman correlation. **D.** Correlation of Cd66+ ARG1+ double staining and ARG1+ CXCR2+ staining in NSCLC, p<0.0001, Spearman correlation.

Supplementary Figure 4:

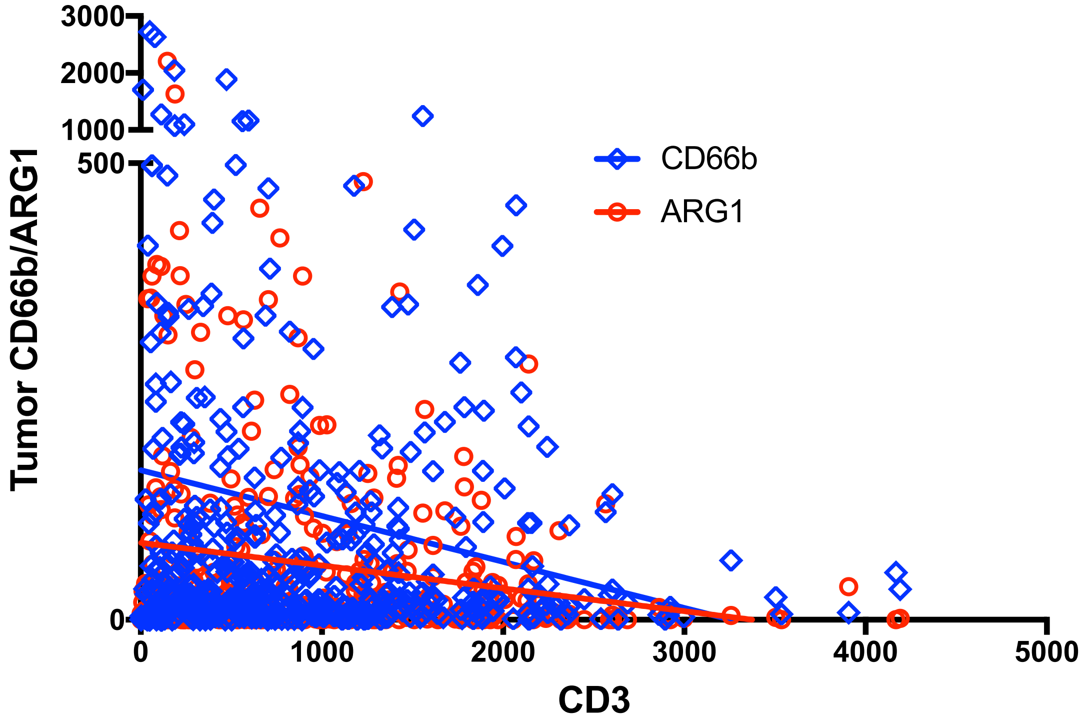

Supplementary figure 4:

Correlation of CD66b, ARG1, and CD3 in multiplex IHC stained Tissue microarray. Correlation of CD3 staining with single CD66b, (p=0.001), or single ARG1 staining (p=0.005), p values were determined by Pearson correlation.

Supplementary Figure 5:

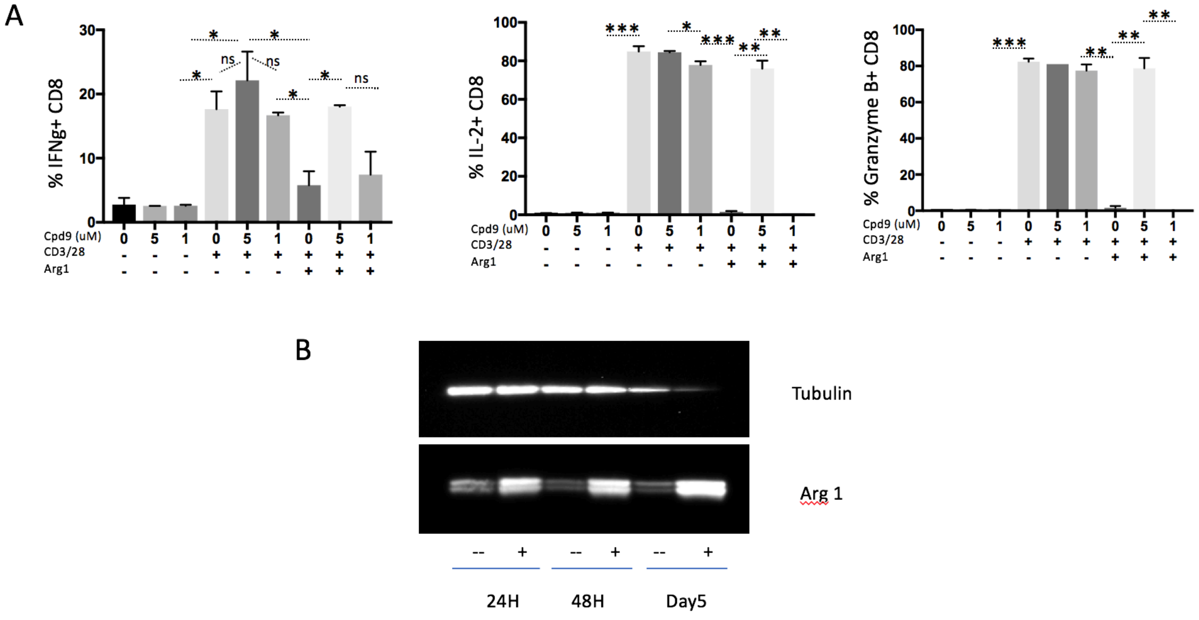

Supplementary figure 5:

Evaluation of the activity of Cpd 9 at different doses and Arg1 expression in peritoneal macrophages.

A. Restoration of IFNg, IL-2 and Granzyme B production of splenocytes in the media containing recombinant arginase (1uM) and varying concentrations of Compound 9. Expression of these molecules were evaluated by intracellular staining followed by flow cytometry. **B.** Arginase 1 expression in peritoneal macrophages (PM). PM cells were treated with IL4/IL10/IL13 (50ng/ml) and PGE2(2.6uM) for 24H, 48H or 5days Protein lysates were prepared and WB performed for Arg1 and tubulin.

Supplementary Figure 6:

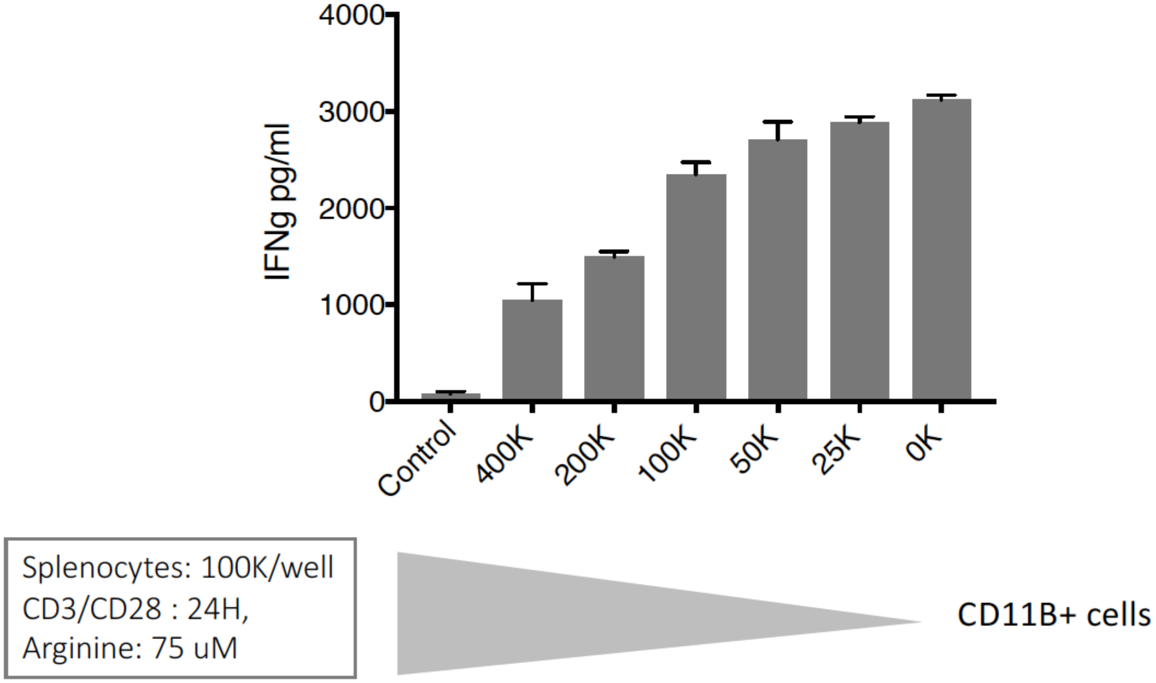

Supplementary Figure 6:

CD11b+ MDSCs sorted from ID8 tumor ascites reduced of IFNg secretion by splenocytes. 400-200-100-50-25-12.5 x10^3 CD11b+ MDSCs cells sorted from ID8 tumor ascites fluid were added to activated splenocytes (CD3/28) in the presence of 75 uM arginine. After 24 hs IFNg was determined by ELISA in the culture supernatants.

Supplementary Figure 7:

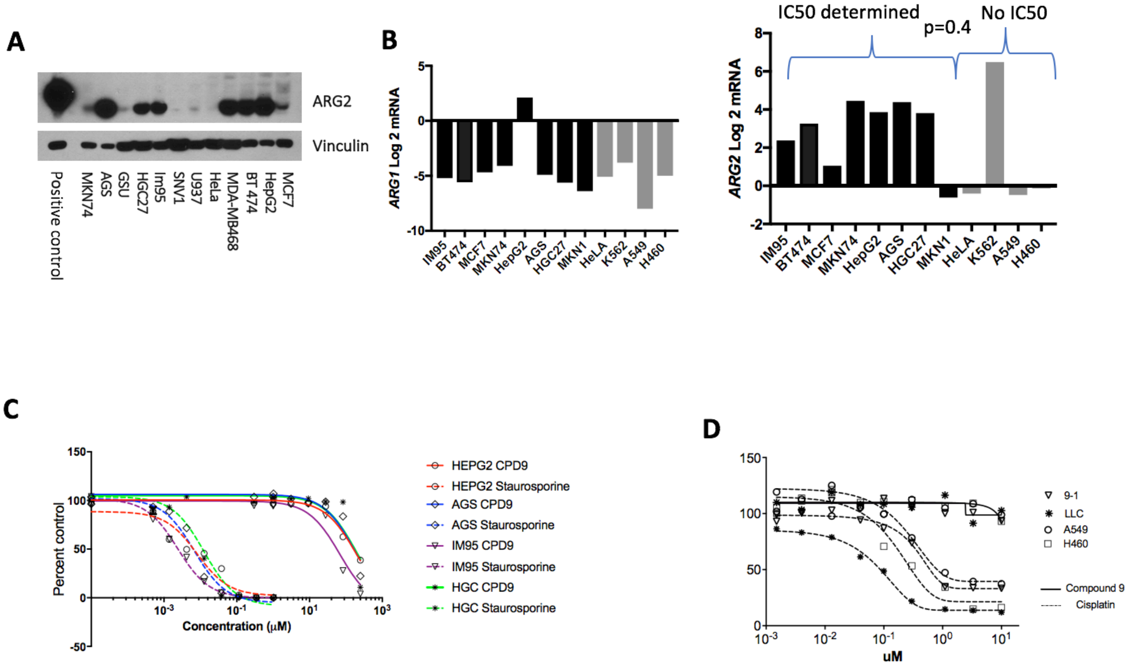

Supplementary Figure 7:

Arginase inhibitor sensitivity correlates with Arginase expression in cancer cell lines.

A. Arginase 2 expression in 12 human cancer cell lines MCF7, HepG2, BT-474, MDA-MB468, HeLa, U937, SNV1, Im95, HGC27, GSU, AGS, MKN74 detected by western blot analysis. **B**. Quantification of ARG1 and ARG2 mRNA levels for Cpd9 sensitive (and IC-50 determined) or not-sensitive (no IC50 determined) cell lines from the cell lines in CCLE dataset.

C. IC50 graph of three Cpd9 sensitive gastric carcinoma cell lines IM-95, AGS, and HGC and liver carcinoma line HepG2 was determined using CTGlo after two days of incubation with varying levels of Cpd9. **D:** In vitro efficacy of Cpd9 in lung cancer cell lines. Growth of murine (LLC, KP9-1 and human (A549, H460) lung cancer lines under various Cpd9 or cisplatin concentrations showing lack of sensitivity to Cpd9.
